# The Role of Heat Shock Protein 70 in the Protective Effect of YC-1 on β-Amyloid-Induced Toxicity in Differentiated PC12 Cells

**DOI:** 10.1371/journal.pone.0069320

**Published:** 2013-07-26

**Authors:** Yung-Chieh Tsai, Yen-Mei Lee, Kwok-Keung Lam, Jui-Fen Lin, Jhi-Joung Wang, Mao-Hsiung Yen, Pao-Yun Cheng

**Affiliations:** 1 Department of Obstetrics and Gynecology, Chi-Mei Medical Center, Tainan, Taiwan; 2 Department of Medicine, Taipei Medical University, Taipei, Taiwan; 3 Department of Sport Management, Chia Nan University of Pharmacy and Science, Tainan, Taiwan; 4 Department of Pharmacology, National Defense Medical Center, Taipei, Taiwan; 5 Department of Pharmacology, Taipei Medical College, Taipei, Taiwan; 6 Department of Anesthesiology, Catholic Mercy Hospital, Hsin-Chu, Taiwan; 7 Department of Medical Research, Chi-Mei Medical Center, Tainan, Taiwan; 8 Department of Chinese Pharmaceutical Sciences and Chinese Medicine Resources, China Medical University, Taichung, Taiwan; Boston University Medical School, United States of America

## Abstract

Neurodegenerative brain disorders such as Alzheimer’s disease (AD) have been well investigated. However, significant methods for the treatment of the progression of AD are unavailable currently. Heat shock protein 70 (Hsp70) plays important roles in neural protection from stress by assisting cellular protein folding. In this study, we investigated the effect and the molecular mechanism of YC-1, an activator of guanylyl cyclase (GC), on Aβ_25–35_-induced cytotoxicity in differentiated PC12 cells. The results of this study showed that Aβ_25–35_ (10 µM) significantly increased p25 protein production in a pattern that was consistent with the increase in μ-calpain expression. Moreover, Aβ_25–35_ significantly increased tau hyperphosphorylation and induced differentiated PC12 cell death. YC-1 (0.5–10 µM) prevented the cell death induced by Aβ_25–35_. In addition, YC-1 (1, 10 µM) significantly blocked Aβ_25–35_-induced μ-calpain expression and decreased the formation of p25 and tau hyperphosphorylation. Moreover, YC-1 (5–20 µM) alone or combined with Aβ_25–35_ (10 µM) significantly increased the expression of Hsp70 in differentiated PC12 cells. The neuroprotective effect of YC-1 was significantly attenuated by an Hsp70 inhibitor (quercetin, 50 µM) or in PC12 cells transfected with an Hsp70 small interfering RNA. However, pretreatment of cells with the GC inhibitor ODQ (10 µM) did not affect the neuroprotective effect of YC-1 against Aβ_25–35_ in differentiated PC12 cells. These results suggest that the neuroprotective effect of YC-1 against Aβ_25–35_-induced toxicity is mainly mediated by the induction of Hsp70. Thus, YC-1 is a potential agent against AD.

## Introduction

Alzheimer’s disease (AD) is the most common cause of dementia in the aged population. AD is characterized by two pathological hallmarks consisting of extracellular plaques of β-amyloid peptide aggregates [1] and intracellular neurofibrillary tangles composed of the hyperphosphorylated microtubular protein tau [2]. The β-amyloid deposition that constitutes the plaques is composed of a 39–42 amino-acid peptide (Aβ) that is the proteolytic product of the amyloid precursor protein (APP) by β/γ secretases. Calpains modulate processes that govern the function and metabolism of key proteins in the pathogenesis of AD, including tau and APP [3]. Cyclin-dependent kinase 5 (cdk5), which promotes the phosphorylation of tau, has been implicated in the pathological processes that contribute to neurodegeneration in AD. p35 is a neuron-specific activator of cdk5, and conversion of p35 into p25 by calpain-dependent proteolysis causes prolonged activation and mislocalization of cdk5. Consequently, the p25/cdk5 kinase hyperphosphorylates tau, disrupts the cytoskeleton, and promotes apoptosis of primary neurons.

Heat shock proteins (Hsps) are the major molecular chaperones that mediate the proper folding of other proteins and ensure that these proteins maintain their native conformations during conditions of stress [4], [5]. In addition, Hsps are required for protein trafficking to target organelles and to facilitate the transfer of misfolded proteins to the proteasome, for degradation [4]. Mammalian Hsps have been classified into families on the basis of their molecular weight, including Hsp27, Hsp40, Hsp60, Hsp70, Hsp90, and Hsp110. These molecular chaperones are either constitutively expressed or inducibly synthesized after cellular stress. Hsp70 chaperones are an important part of the cellular protein quality control and degradation systems [6], [7]. The Hsp70 family includes the heat shock cognate protein Hsc70 and the heat shock protein Hsp70. Studies demonstrated the presence of elevated levels of Hsp70 synthesis and accumulation in AD brain [8] and neurons with strong staining for Hsp70 that did not contain neurofibrillary tangles [9]. Induction of Hsp70 by heat preconditioning protected against AD-like hyperphosphorylation of tau in PC12 cells [10], and induction of Hsp70 by geldanamycin reduced okadaic acid-induced tau phosphorylation and aggregation in COS-1 cells expressing human tau [9]. These findings suggest that Hsp70 represents an important molecular target for neuroprotective strategies in AD treatment.

YC-1 [3-(50-hydroxymethyl-20-furyl)-1-benzylindazole] is a synthetic benzylindazole compound originally developed as an activator of guanylyl cyclase (GC) to inhibit platelet aggregation and vascular contraction [11]. Several lines of evidence have shown that YC-1 exhibits therapeutic potential for the treatment of a series of vascular diseases, including hypertension, thrombosis, erectile dysfunction, and postangioplasty restenosis [12], [13]. Recent studies revealed that YC-1 induces Hsp70 expression and prevents oxidized LDL-mediated apoptosis in vascular smooth muscle cells [14]. Thus, the aim of this study was to determine whether YC-1 can prevent Aβ-induced cytotoxicity in PC12 cells and whether the neuroprotective effect of YC-1 is mediated by the induction of Hsp70.

## Materials and Methods

### Cell Culture

A PC12 cell line (derived from the American Type Culture Collection, CRL-1721) purchased from the Food Industry Research and Development Institute, Hsinchu, Taiwan and was cultured in RPMI 1640 medium (Gibco-BRLTM, Gaithersburg, MD, USA) supplemented with 10% horse serum (v/v; Gibco-BRLTM), 5% fetal bovine serum (v/v; Gibco-BRLTM), and 0.1% gentamicin (v/v; Gibco-BRLTM). Cells were cultured on 75 cm flasks. PC12 cells were maintained in a 37°C incubator in a water-saturated, 5% CO_2_ atmosphere. The cells were subcultured when the cultures were 80–90% confluent (split ratio, 1∶4). The medium was refreshed approximately three times a week. PC12 cells were induced to differentiate by plating 2.5×10^6^ cells on 10% collagen-coated 100 mm dishes and cultured in RPMI 1640 medium supplemented with 10% horse serum (v/v), 5% fetal bovine serum (v/v), 0.1% gentamicin (v/v), and 25 ng/mL nerve growth factor (NGF; Sigma, St. Louis, MO, USA) (differentiation medium, DM) for 48 h before all experiments performed in the present study.

### Aβ_25–35_ Preparation and Treatment

β-Amyloid (Aβ), a 39–43-amino-acid β-sheet peptide, aggregates in the brain to form the major component of characteristic deposits known as senile plaques [15]. Of all the Aβ derivatives studied so far, Aβ_25–35_ (GSNKGAIIGLM) is the shortest fragment that exhibits large β-sheet fibrils and retains the toxicity of the full-length peptide [16]. It has been proposed that Aβ_25–35_ represents the biologically active region of Aβ. *In vitro* studies have shown that, unlike the full-length peptide, it does not require aging to aggregate and become toxic [17]. Therefore, in this study, Aβ_25–35_ was employed as a neurotoxicant and the neuroprotective effect of YC-1 against Aβ_25–35_-induced cytotoxicity was determined by measuring the viability of differentiated PC12 cells after incubation with Aβ_25–35_ in the absence or presence of YC-1 using an MTT assay. The Aβ_25–35_ peptide (Sigma) was dissolved in sterile deionized water and the stock solution (1.0 mM) was stored in aliquots at –20°C. Aβ_25–35_ was added to the cultures at 96 h of differentiation to a final concentration of 1–20 µM. Control and Aβ_25–35_-treated cells were cultured in DM for an additional 24 h, unless otherwise noted.

### Assessment of Cell Viability

Cell survival was evaluated by MTT (3-(4,5-dimethylthiazol-2-yl)-2,5- diphenyltetrazolium bromide) reduction. Cells were incubated with various concentrations of Aβ_25–35_ and/or YC-1 for 24 h in a 12-well plate. MTT reduction was started by adding MTT solution (0.5 µg/mL) per well. Plates were incubated at 37°C. After 3 h incubation, the reaction was stopped by adding 200 µL of isopropranol. Absorbance was determined at 570 nm and the reference wave was determined at 630 nm using a microplate reader (Model 550, BIO-RAD Laboratories, CA, USA) [18].

### siRNA Transfection

An anti-Hsp70 siRNA was chemically synthesized by Invitrogen (Carlsbad, CA, USA). The following sequences were used: anti-Hsp70 sense, 5′–UUA CCU GGC UCU UUG CUG CUG CUC C–3′; anti-Hsp70 antisense, 5′–GGA GCA GCA GCA AAG AGC CAG GUA A–3′. siRNA was complexed with Lipofectamine™ RNAiMAX (Invitrogen) according to the manufacturer’s instructions. Cells plated in 6-well culture plates and transfection complexes that contained Neurobasal medium without supplements and the complex of Lipofectamine™ RNAiMAX and 40 nM siRNA. In addition, Tau siRNA (sc-36615; Snata Cruz Biotechnology) is a pool of three target specific siRNAs designed to knock down gene expression. Tau siRNA were transfection to cells according to the manufacturer’s instructions. Cells were transfected with siRNA for at least 36 h at 37°C. The cultures treated with agents and subjected to protein analyses or cell viability assay.

### Preparation of Total Cellular Proteins

PC12 cell lysates were prepared using 20 mM Tris–HCl, pH 7.4, 150 mM NaCl, 1% Triton X-100, 2.5 mM EDTA, 2.5 mM EGTA, and 1∶200 protease inhibitor cocktail set III (Calbiochem, La Jolla, CA, USA). Lysates were kept on ice for 30 min and centrifuged at 10,000×*g* for 10 min at 4°C. Protein concentration was determined using the BCA protein assay kit (Pierce, Rockford, IL, USA) according to the manufacturer’s instructions.

### Western Blot Analysis

Sodium dodecyl sulfate–polyacrylamide gel electrophoresis (SDS–PAGE) was performed according to standard procedures. Samples containing 20–40 µg of proteins from PC12 cells were electrophoresed and then transferred to nitrocellulose membranes (Millipore, Bedford, MA, USA). The nitrocellulose membrane was cut according the molecular weight of protein and be incubated with different protein antibody. Therefore, one result of different proteins could get in one nitrocellulose membrane. Meanwhile, these proteins had the same internal standard (β-actin). Immunoblotting was carried out as described previously [19] using a rabbit anti-calpain antibody (1∶1000; Cell Signaling Technology), a rabbit anti-p35/25 antibody (1∶1000; Cell Signaling Technology), a mouse anti-total tau antibody (1∶1000; BD Pharmingen, USA), an anti-tau phospho S199/S202 antibody (1∶1000; GeneTex, Inc.), a mouse anti-Hsp70 antibody (1∶1000; Stressgen, USA), and a mouse anti-β-actin monoclonal antibody (1∶5000; Sigma, St. Louis, MO, USA). The appropriate peroxidase-conjugated secondary antibodies were used, detection was performed using an enhanced chemiluminescence kit (Pierce), and the membranes were exposed to X-ray film (Kodak, Rochester, NY, USA) for 5 min. Relative abundance of different protein compared with beta-actin. All data are means ± SD of five independent observations with different cell passages and on different days. The density of the respective bands was quantified by densitometric scanning of the blots using the Image-Pro software (Media Cybermetrics, Inc.).

### Statistical Analyses

Data are expressed as the mean ± SD. Analysis of variance (ANOVA) followed by a Newman–Keuls test was used for statistical comparisons. *P*<0.05 was considered significant.

## Results

### Effects of YC-1 on the Cell Toxicity Induced by Aβ_25–35_ in Differentiated PC12 Cells

**Figure 1:**
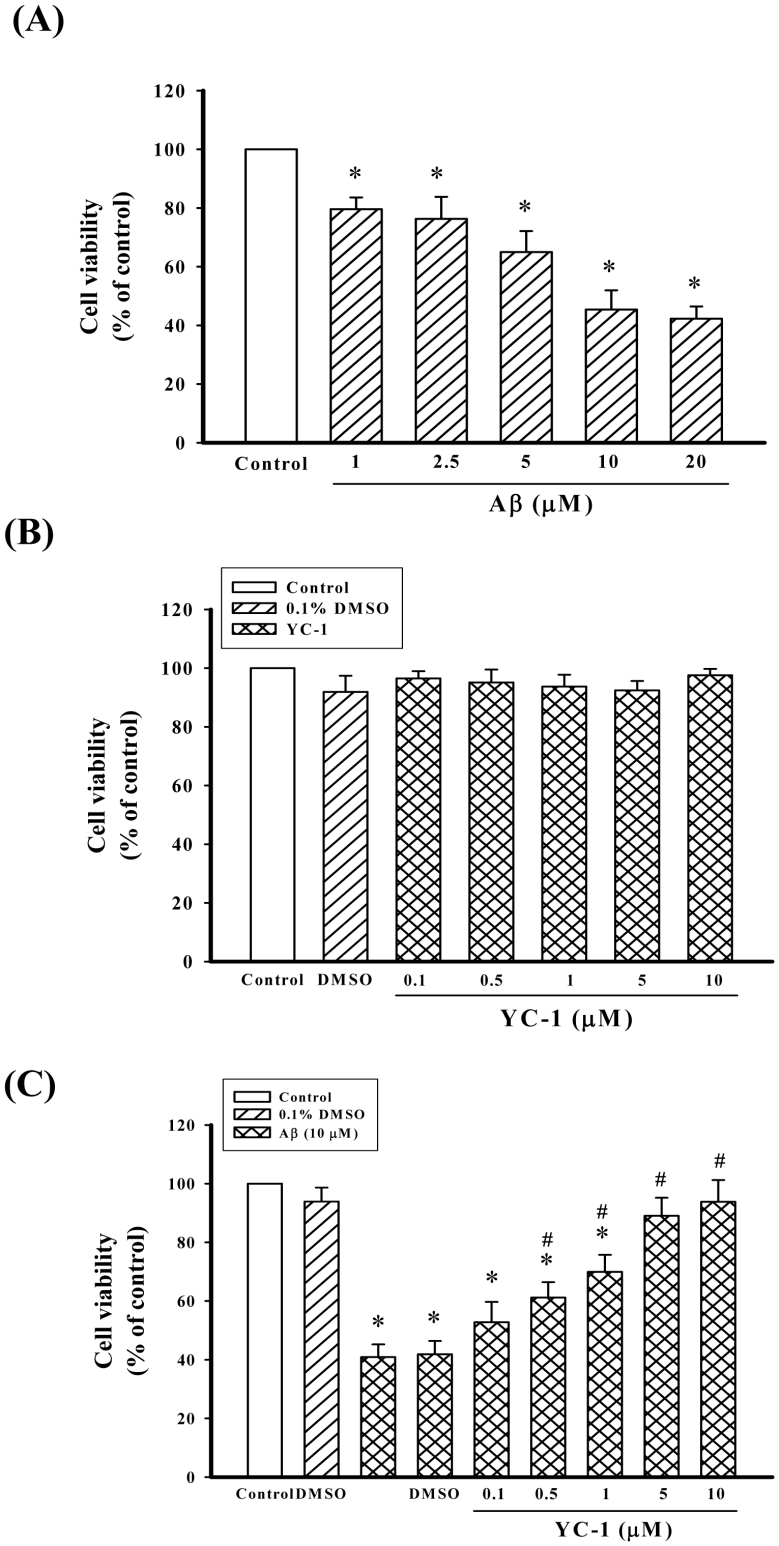
YC-1 prevented the death of differentiated PC12 cells induced by Aβ_25–35_. (A and B) Concentration-dependent effect of Aβ_25–35_ and YC-1 on the viability of differentiated PC12 cells. Differentiated PC12 cells were treated with Aβ_25–35_ (1–20 µM) or YC-1 (0.1–10 µM) for 24 h. (C) Effect of YC-1 on Aβ_25–35_-induced cytotoxicity in differentiated PC12 cells. Differentiated PC12 cells were incubated with different concentrations of YC-1 (0.1–10 µM) in the presence of Aβ_25–35_ (10 µM) for 24 h and cell viability was estimated using an MTT assay. All data shown represent the mean ± SD (n  = 5). *P<0.05 vs. control; ^#^ *P*<0.05 vs. Aβ_25–35_-treated cells.

As shown in Figure 1A, Aβ_25–35_-induced neurotoxicity was concentration dependent in the range of 1–20 µM and cell survival (expressed as the percentage of control measured in the absence of Aβ_25–35_) decreased by ∼40%. Treatment with 10 µM Aβ_25–35_ induced ∼50% cell death; therefore, 10 µM Aβ_25–35_ was used in this study. Treatment with YC-1 alone (0.1–10 µM) did not decrease cell viability compared with DMSO-treated controls (Fig. 1B). However, pretreatment of cells with YC-1 (0.5–10 µM) for 30 min led to a significant reduction in Aβ_25–35_-induced cell toxicity (Fig. 1C).

### Phosphorylated Tau is Mediating the Neurotoxicity of Aβ_25–35_ in Differentiated PC12 Cells

**Figure 2:**
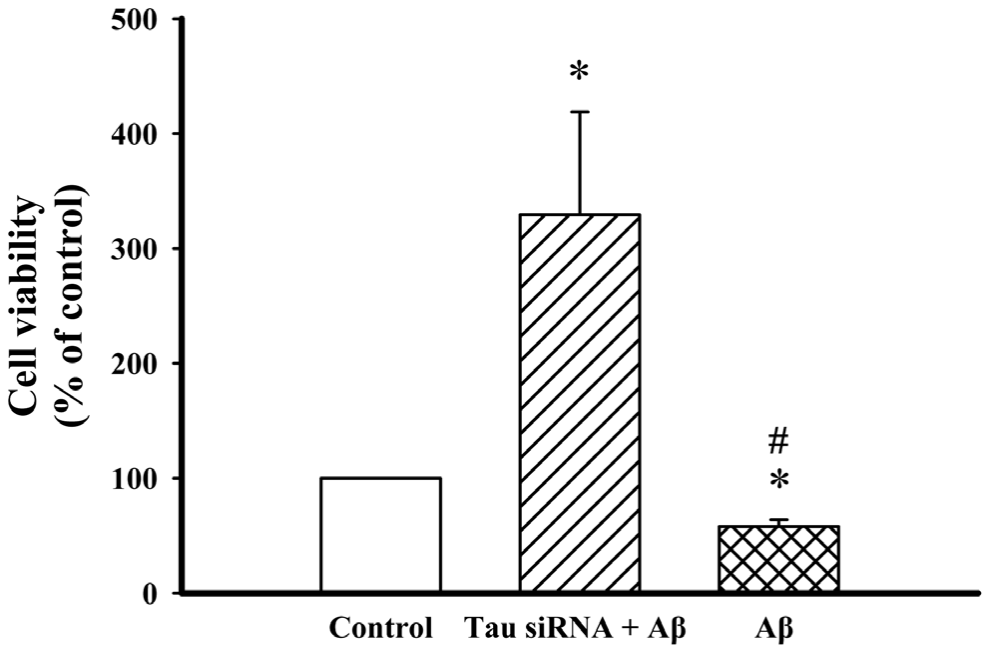
Phosphorylated tau is mediating the nerutoxicity of Aβ_25–35_ in differentiated PC12 cells. Cell viability was estimated using an MTT assay in differentiated PC12 cells transfected with a tau siRNA for 36 h and treated with Aβ_25–35_ (10 µM). All data shown represent the mean ± SD (n  = 3). *P<0.05 vs. control; ^#^ *P*<0.05 vs. cells treated similarly with tau siRNA transfection.

In order to confirm the phosphorylated tau in mediating the neurotoxicity of Aβ_25–35_ in differentiated PC12 cells_,_ the tau siRNA were used to silencing the endogenous tau. As shown in Figure 2, the cell viability was significantly increased in tau siRNA-transfected differentiated PC12 cells after treated with Aβ_25–35_.

### Effects of YC-1 on μ-calpain Protein Expression in Differentiated PC12 Cells

**Figure 3:**
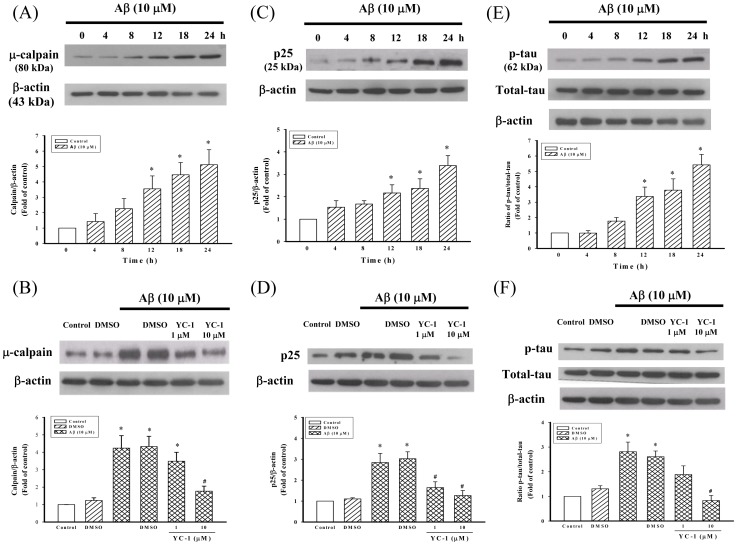
YC-1 inhibited Aβ_25–35_-induced μ-calpain, p25, and phosphorylated tau protein expression. Effects of Aβ_25–35_ (10 µM) on the time-course changes in the expression of (A) μ-calpain, (C) p25, and (E) phosphorylated tau (p-tau) in differentiated PC12 cells. Differentiated PC12 cells were treated with Aβ_25–35_ (10 µM) for 24 h. All data shown represent the mean ±SD (n  = 5). *P<0.05 vs. control. Effects of YC-1 (1, 10 µM) on the expression of (B) μ-calpain, (D) p25, and (F) p-tau induced by Aβ_25–35_ (10 µM) in differentiated PC12 cells. Differentiated PC12 cells were incubated with Aβ_25–35_ (10 µM) and YC-1 (10 µM) was added to the medium 30 min before the addition of Aβ_25–35_. All data are means ± SD of five independent observations with different cell passages and on different days. Depicted are a typical display of protein production (upper panel) and the statistical analysis of the changes of protein (lower panel). The typical data (β-actin) obtain from the same nitrocellulose membrane in Figures 3D and 3F. *P<0.05 vs. control; ^#^ *P*<0.05 vs. Aβ_25–35_-treated cells.

Whether Aβ_25–35_ treatment results in calpain activation, the differentiated PC12 cells were incubated with Aβ_25–35_ (10 µM) for 24 h. As shown in Figure 3A, Aβ_25–35_ produced a time-dependent increase in the expression of the μ-calpain protein (75 kDa) in differentiated PC12 cells. However, the expression of μ-calpain was significantly decreased in differentiated PC12 cells pretreated with YC-1(1, 10 µM) compared with cells treated with Aβ_25–35_ alone (Fig. 3B).

### Effects of YC-1 on p25 Protein Expression in Differentiated PC12 Cells

The cytosolic p25 protein is generated through the proteolytic cleavage of p35 by activated calpain. We investigated whether p25 was upregulated in Aβ_25–35_-treated cells. As illustrated in Figure 3C, p25 protein levels were markedly increased after treatment with Aβ_25–35_ (10 µM) for 12–24 h in differentiated PC12 cells. In contrast, YC-1 (1, 10 µM) pretreatment dramatically reduced the expression of the p25 protein in differentiated PC12 cells compared with cells treated with Aβ_25–35_ alone (Fig. 3D).

### YC-1 Decreased the Tau Hyperphosphorylation that was Induced by Aβ_25–35_ in Differentiated PC12 Cells

Tau hyperphosphorylation and accumulation as neurofibrillary tangles are one of the pathological hallmarks of AD. It is known that hyperphosphorylated tau itself can induce neuronal death and that an increase in tau phosphorylation can be induced by an increase in calcium influx in response to Aβ_25–35_. To assess the effect of Aβ_25–35_ and YC-1 on tau hyperphosphorylation in differentiated PC12 cells, an anti-phosphorylated tau (pS199pS202) antibody was used. As shown in Figure 3E, Aβ_25–35_ (10 µM) induced hyperphosphorylation of the tau protein in time-dependent manner. However, treatment with YC-1 (10 µM) prior to Aβ_25–35_ significantly decreased the amount of tau phosphorylation in differentiated PC12 cells (Fig. 3F).

### The Effect of ODQ on the Protective Role of YC-1: Prevention of Aβ25–35-induced Signaling in Differentiated PC12 Cells

**Figure 4:**
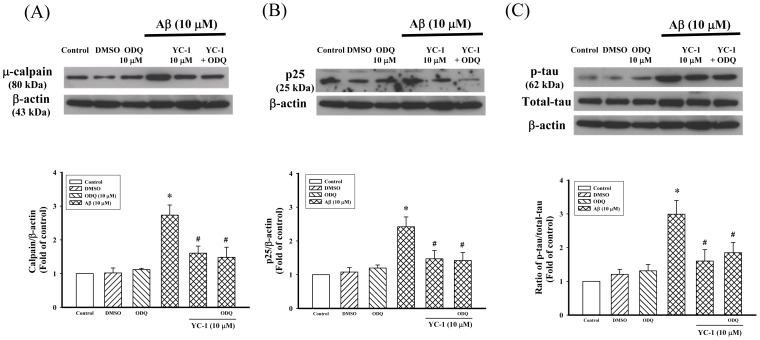
Guanylyl cyclase was not involved in the inhibitory effects of YC-1 in differentiated PC12 cells. Differentiated PC12 cells were incubated with YC-1 (10 µM) in the presence of Aβ_25–35_ (10 µM) for 24 h and ODQ (10 µM; a guanylyl cyclase inhibitor) was added to the medium 30 min before the addition of YC-1. The expression of (A) μ-calpain, (B) p25, and (C) p-tau was determined by Western blotting. All data are means ± SD of five independent observations with different cell passages and on different days. Depicted are a typical display of protein production (upper panel) and the statistical analysis of the changes of protein (lower panel). The typical data (β-actin) obtain from the same nitrocellulose membrane. *P<0.05 vs. control; ^#^ *P*<0.05 vs. Aβ_25–35_-treated cells.

It is well known that YC-1 is an NO-independent soluble guanylyl cyclase (sGC) activator. Here, we investigated whether sGC is involved in the inhibition of Aβ_25–35_-induced μ-calpain, p25 expression, and tau hyperphosphorylation in differentiated PC12 cells. As shown in Figure 4, the sGC inhibitor ODQ (10 µM) did not antagonize the inhibitory action of YC-1 on Aβ_25–35_-induced μ-calpain and p25 expression, as well as tau hyperphosphorylation in differentiated PC12 cells. Therefore, the protective action of YC-1 may not be exerted via an sGC-dependent pathway.

### Effects of Aβ_25–35_ and YC-1 on the Expression of Hsp70 in Differentiated PC12 Cells

**Figure 5:**
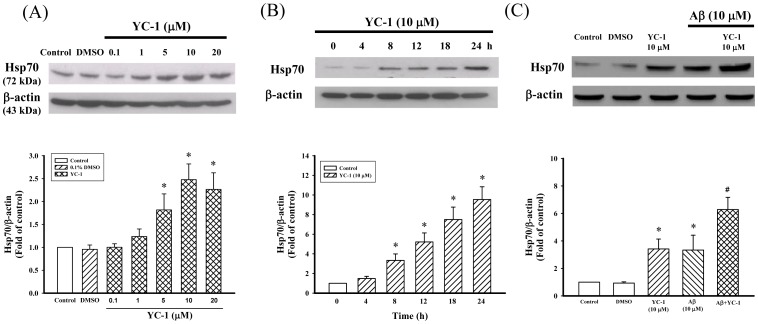
YC-1 enhanced the expression of Hsp70 in differentiated PC12 cells. (A) Effect of YC-1 (0.1–20 µM) on the expression of Hsp70 in differentiated PC12 cells. Differentiated PC12 cells were incubated with different concentrations of YC-1 (0.1–20 µM) for 24 h and the expression levels of Hsp70 were determined by Western blotting. (B) Effect of YC-1 (10 µM) on the time-course changes in the expression of Hsp70 in differentiated PC12 cells. (C) Effect of YC-1 (10 µM) alone and/or with Aβ_25–35_ (10 µM) on the expression of Hsp70 in differentiated PC12 cells. Differentiated PC12 cells were incubated with YC-1 (10 µM) in the presence of Aβ_25–35_ (10 µM) for 24 h. All data shown represent the mean ± SD (n  = 5). *P<0.05 vs. control; ^#^ *P*<0.05 vs. Aβ_25–35_-treated cells.

We evaluated whether YC-1 increased Hsp70 expression in differentiated PC12 cells. As shown in Figure 5A and B, Hsp70 constitutively expressed in vehicle-treated subjects and was upregulated in a concentration-dependent manner after YC-1 (5–20 µM) treatment (Fig. 5A). In addition, we evaluated the variation of Hsp70 expression after YC-1 (10 µM) treatment. Results showed that Hsp70 expression increased at 8 h and reached a peak at 18 and 24 h after YC-1 treatment (Fig. 5B). Furthermore, Aβ_25–35_ alone induced the expression of Hsp70 in differentiated PC12 cells and YC-1 together with Aβ_25–35_ led to a more significant increase in the expression of Hsp70 (Fig. 5C).

### Involvement of Hsp70 in the Neuroprotective Effect of YC-1 in Differentiated PC12 Cells

**Figure 6:**
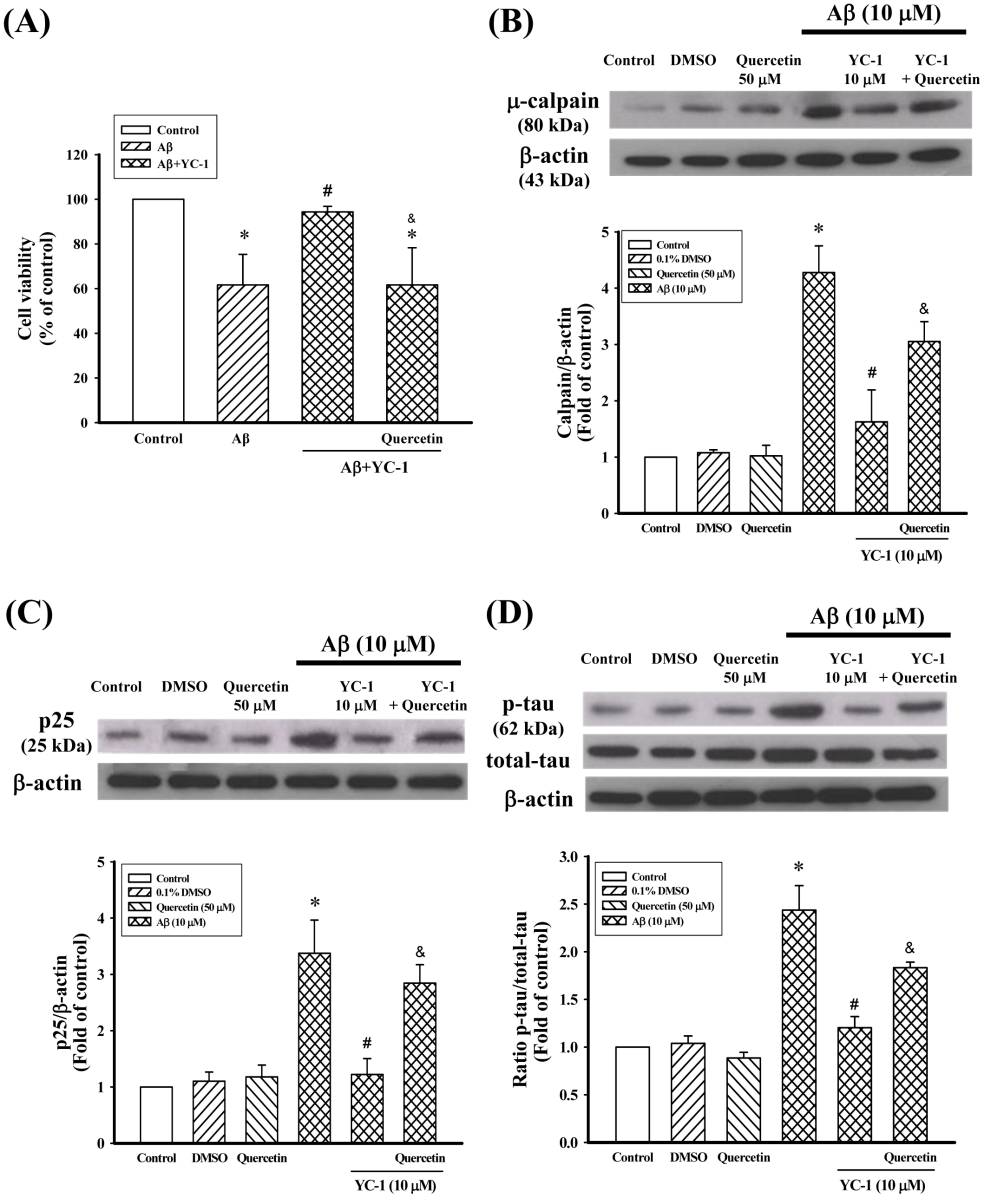
Hsp70 was involved in the inhibitory effects of YC-1 in differentiated PC12 cells. Differentiated PC12 cells were incubated with YC-1 (10 µM) in the presence of Aβ_25–35_ (10 µM) for 24 h and quercetin (50 µM; an Hsp70 inhibitor) was added to the medium 30 min before the addition of YC-1. (A) Cell viability was estimated using an MTT assay in differentiated PC12 cells. The expression of (B) μ-calpain, (C) p25, and (D) p-tau was determined by Western blotting. All data shown represent as the mean ± SD (n  = 5). *P<0.05 vs. control; ^#^ *P*<0.05 vs. Aβ_25–35_-treated cells; ^&^ *P*<0.05 vs. Aβ_25–35_/YC-1-treated cells.

**Figure 7:**
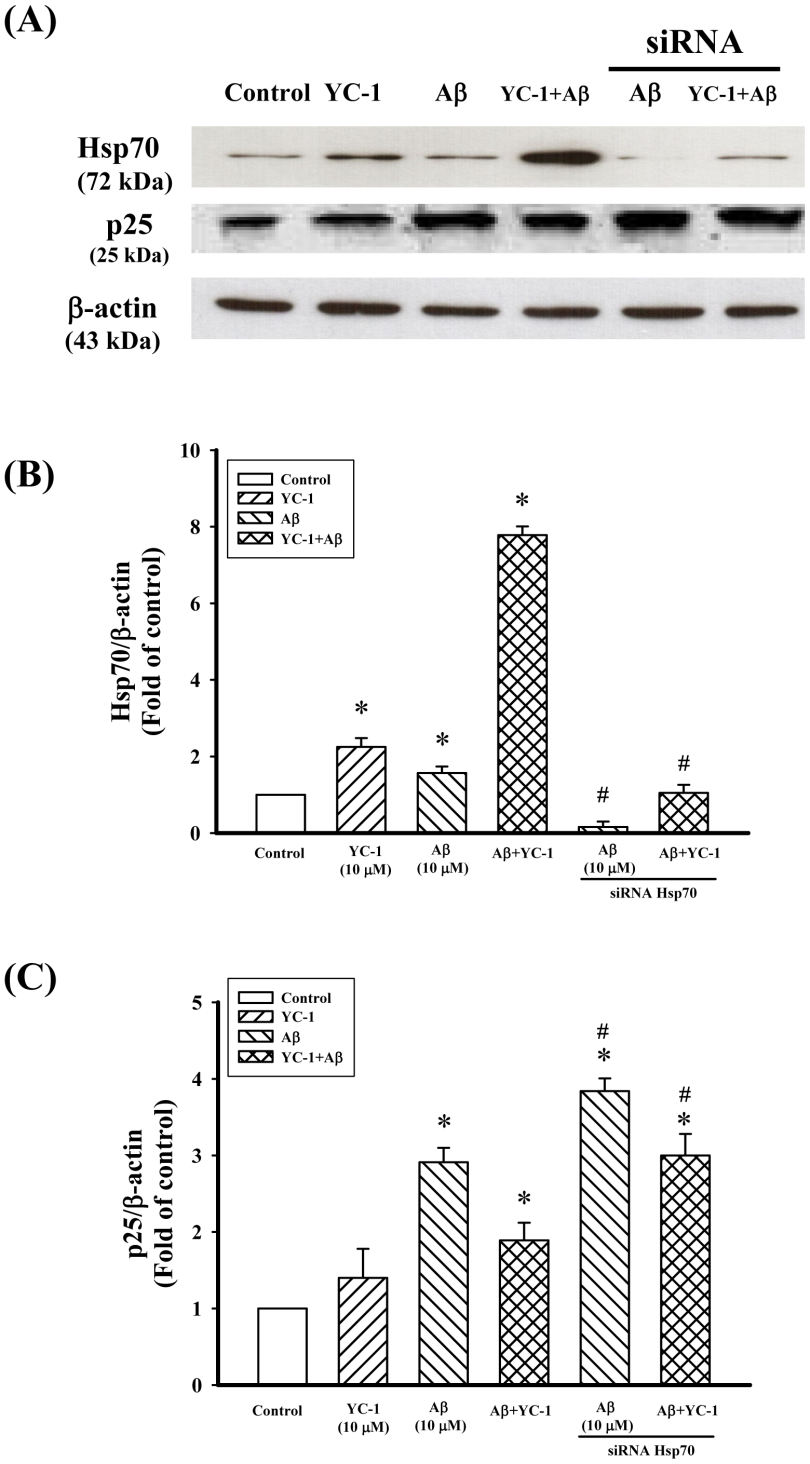
Hsp70 inhibition attenuated the neuroprotection mediated by YC-1 in differentiated PC12 cells. Western blotting detection of the expression of Hsp70 and p25 in differentiated PC12 cells transfected with an Hsp70 siRNA for 36 h and treated with Aβ_25–35_ (10 µM) and YC-1 (10 µM). Representative Western blotting data are shown in (A). (B) and (C) are bar graphs of the expression of Hsp70 and p25, respectively. All data shown represent the mean ± SD (n  = 3). *P<0.05 vs. control; ^#^ *P*<0.05 vs. cells treated similarly but without Hsp70 siRNA transfection.

To assess whether Hsp70 is involved in the inhibitory effect of YC-1 on Aβ_25–35_, the Hsp70 inhibitor quercetin was used. As shown in Figure 6A, quercetin (50 µM) significantly counteracted the neuroprotective effect of YC-1 in differentiated PC12 cells after treated with Aβ_25–35_. Meanwhile, quercetin significantly antagonized the inhibitory action of YC-1 on Aβ_25–35_-induced μ-calpain expression, p25 expression, and tau hyperphosphorylation in differentiated PC12 cells (Fig. 6B–6D). Moreover, quercetin alone did not have any effects on the expression of μ-calpain, expression of p25, or tau hyperphosphorylation in differentiated PC12 cells. Furthermore, the expression of the Hsp70 protein in YC-1-treated cells was knocked down by Hsp70 siRNA, and the inhibitory action of YC-1 on Aβ_25–35_-induced p25 expression was markedly attenuated in Hsp70-siRNA-transfected differentiated PC12 cells (Fig. 7).

## Discussion

The present study provides insights into the role of Hsp70 and the molecular mechanism via which YC-1 protects neurons from the toxicity associated with Aβ_25–35_ peptides. Although YC-1 attenuated cell death in Aβ_25–35_-treated differentiated PC12 cells, it also decreased Aβ_25–35_-induced μ-calpain and p25 expression levels, as well as tau hyperphosphorylation. In addition, YC-1 alone or combined with Aβ_25–35_ significantly increased the expression of Hsp70 in differentiated PC12 cells. Moreover, the inhibitory effect of YC-1 on Aβ_25–35_ was significantly attenuated by pretreatment with the Hsp70 inhibitor quercetin and/or Hsp70 siRNA. These results suggest that Hsp70 plays an important role in the neuroprotective effect of YC-1 in differentiated PC12 cells.

Intracellular aggregates of hyperphosphorylated tau, as neurofibrillary tangles, are one of the pathological hallmarks of AD. A previous study demonstrated that tau is essential for Aβ-induced neurotoxicity [20]. In addition, several studies have indicated that Aβ increases the hyperphosphorylation of tau at disease-relevant sites [21]–[25] and induces subsequent tau aggregation into paired helical filament-like filaments [26]. Furthermore, hyperphosphorylated tau induces neurotoxicity downstream of Aβ [27]. Significant increase in tau hyperphosphorylation was reported in postmortem tissues obtained from the brains of AD patients [28]. These findings indicate that the prevention of tau hyperphosphorylation may protect cells against Aβ-induced neurotoxicity. The results of the current study confirmed that YC-1 decreased the level of the tau hyperphosphorylation induced by Aβ_25–35_ (Fig. 3F). This is the first experiment to demonstrate that the neuroprotective effect of YC-1 against Aβ_25–35_ occurs via the inhibition of tau hyperphosphorylation.

Numerous studies have shown an abnormal activation of the calpain system in the brain of AD patients [29]–[31]. In addition, calpain activation has been implicated in the cleavage of a number of other proteins that are relevant to AD, including APP, p35, and microtubule-associated proteins [32]–[35]. Activated calpain cleaves the normal regulatory subunit p35 to p25, thus forming a p25/cdk5 complex with an activity profile that is substantially higher compared with that of p35 associated with the kinase [36]. Consequently, the p25/cdk5 kinase hyperphosphorylates tau, disrupts the cytoskeleton, and promotes the apoptosis of neurons. In this study, treatment of differentiated PC12 cells with Aβ_25–35_ decreased cell viability (Fig. 1C). Moreover, the expression of μ-calpain and p25 was significantly increased after a challenge with Aβ_25–35_ (Fig. 3A, C, and E). In contrast, YC-1 significantly attenuated Aβ_25–35_- induced μ-calpain activation, p25 protein expression, and cell death (Fig. 3B, D, and F). These data suggest that YC-1 has a great potential as a new therapeutic agent for AD.

It is well known that YC-1 is an activator of GC. To understand better the molecular mechanism underlying this phenomenon, we investigated the GC–cGMP pathway in differentiated PC12 cells after treatment with Aβ_25–35_ and YC-1. The inhibitory effects of YC-1 on the Aβ_25–35_-induced μ-calpain and p25 protein expression, as well as on tau hyperphosphorylation, in differentiated PC12 cells were examined in the presence of ODQ, an inhibitor of GC. The results (Fig. 4) revealed that ODQ did not modulate the inhibitory effects of YC-1 in differentiated PC12 cells. In addition, YC-1 did not affect the increase of intracellular calcium level caused by Aβ_25–35_ (data not shown)_._ Taken together, these results indicated that YC-1 attenuates Aβ-induced cytotoxicity in a cGMP-independent manner.

It is well established that Hsps represent an important cellular protective mechanism against a variety of stresses and insults [37], [38]. The cellular protection provided by Hsps is attributed to their molecular chaperone function, as it facilitates nascent protein folding and refolding and the degradation of abnormally folded proteins [38], [39]. A large body of evidence indicates that Hsps are potent suppressors of neurodegeneration and are, therefore, promising therapeutic targets for neurodegenerative disorders [40], [41]. In this study, YC-1 alone or together with Aβ_25–35_ significantly increased the expression of the Hsp70 protein in differentiated PC12 cells (Fig. 5). In addition, the inhibitory effect of YC-1 on Aβ_25–35_-induced μ-calpain activation, p25 protein expression, and tau phosphorylation were significantly attenuated by pretreatment with the Hsp70 inhibitor quercetin and/or Hsp70 siRNA (Figs 6 and 7). These results indicate that the neuroprotective effect of YC-1 against Aβ_25–35_ occurs via the induction of the expression of Hsp70. Although we have no direct evidence of a physical interaction between Aβ_25–35_ and Hsp70, it is plausible that Hsp70 is critical in the sequestration of intraneuronal Aβ [42]. The potential mechanism underlying the YC-1-mediated induction of Hsp70 expression was not elucidated in this study. However, YC-1 has been found to increase the expression of heat shock factor-1 in vascular smooth muscle cells [14]. Further studies are needed to clarify the mechanism via which YC-1 regulates the expression of Hsp70.

In conclusion, the present study examined the neuroprotective effect of YC-1 in differentiated PC12 cells in an *in vitro* model of AD. YC-1 suppressed Aβ_25–35_ toxicity via the inhibition of Aβ_25–35_-induced calpain activation, leading to decreased p25 formation and subsequent tau hyperphosphorylation. Moreover, the induction of the expression of Hsp70 may be involved in the neuroprotective effect of YC-1 in differentiated PC-12 cells. These findings suggest that YC-1 may be a potential agent for the treatment of AD.

## Supporting Information

Figure S1 **The raw data for the** **Figure 3D, 3F** **, and** **Figure 4** **.** Protein samples from PC12 cells were electrophoresed and then transferred to nitrocellulose membranes. The nitrocellulose membrane was cut according the molecular weight of protein and be incubated with different protein antibody. Therefore, one result of different proteins could get in one nitrocellulose membrane. Meanwhile, these proteins had the same internal standard (β-actin).
(PDF)
