# Supplementary material for: The Role of Heat Shock Protein 70 in the Protective Effect of YC-1 on β-Amyloid-Induced Toxicity in Differentiated PC12 Cells
Source: PLoS One. 2013 Jul 26;8(7):e69320. doi: 10.1371/journal.pone.0069320 (PMC3724837; doi:10.1371/journal.pone.0069320)

2/26

β-actin

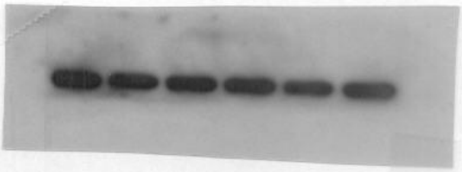

Calpain 1° 1:500 2° 1:5000

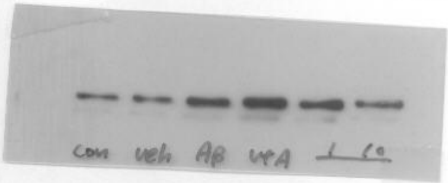

pHF – tau 1° 1:500 2° 1:5000

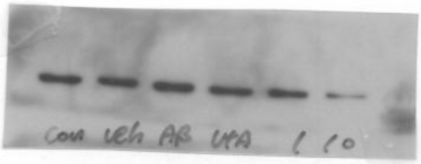

p25

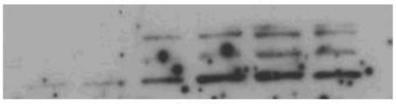

total – tau 1° 1:1000 2° 1:5000

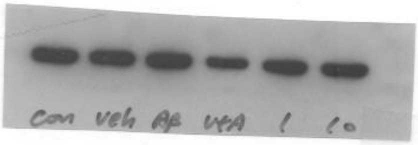

CDK5 1° 1:1000 2° 1:5000

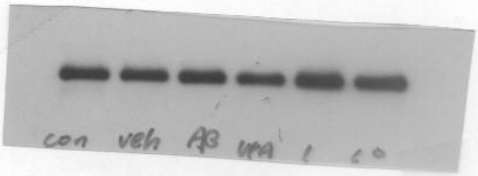

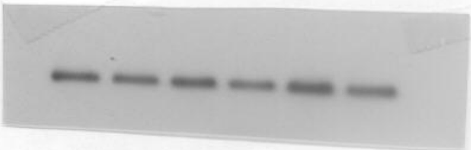

1/7 total

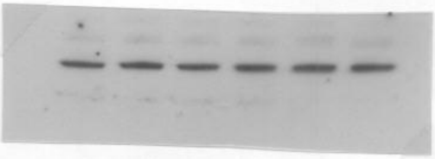

3/1

β-actin

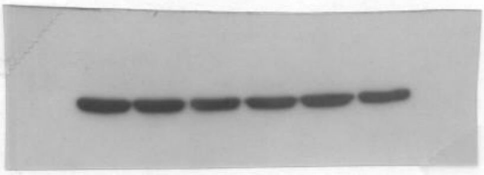

calpain

p35

1° 1:12000 2° 1:8000

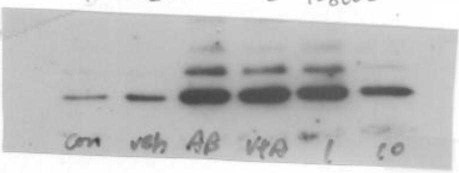

CDK5 1° 1:1000 2° 1:5000

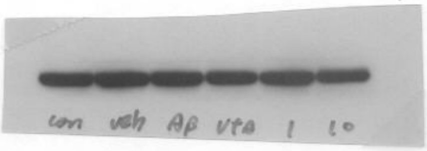

HSP70 1° 1:1000 2° 1:3000

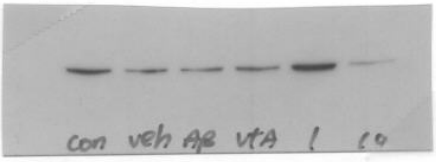

↑
x

PHF-tau 1° 1:1000 2° 1:5000

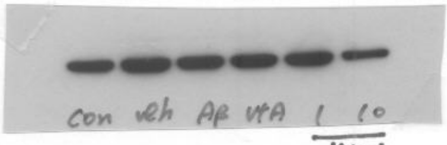

YC-1

total-tau 1° 1:1000 2° 1:5000

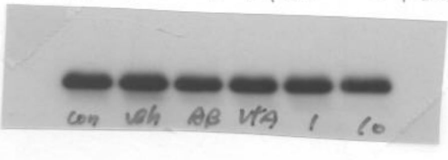

3/2 -1

β-actin

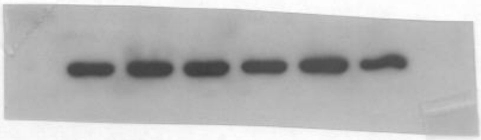

calpain

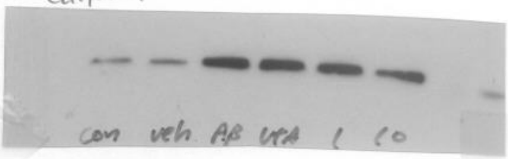

PHF

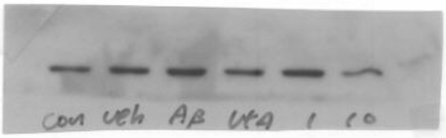

p35

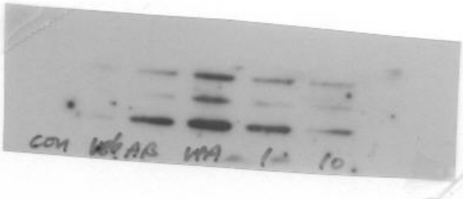

total-tau

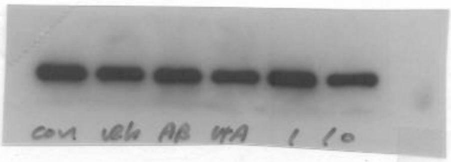

CDK5

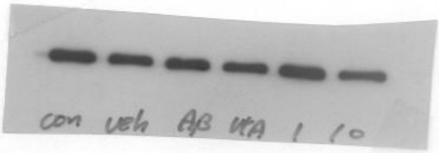

3/2 -2

β-actin

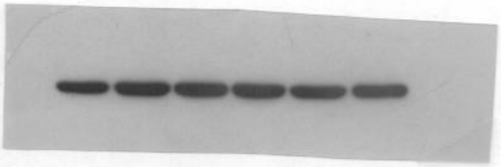

HSP70 1° 1:1000 2° 1:5000

Calpain

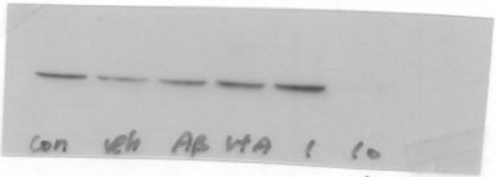

↑
X

p25/35

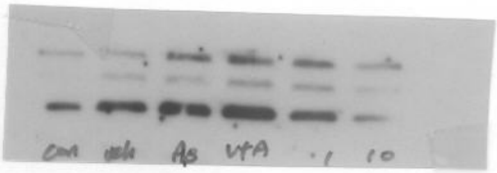

PHF-tau 1° 1:1000 2° 1:5000

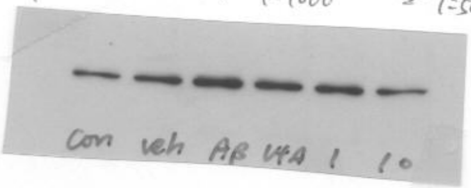

CDK5

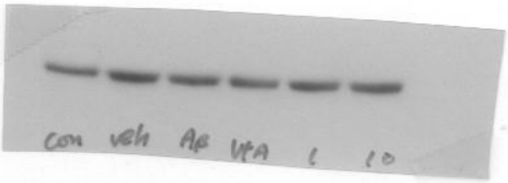

Total-tau 1° 1:1000 2° 1:5000

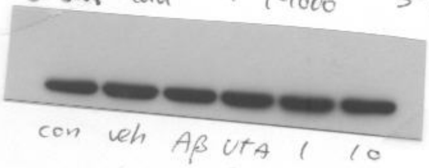

con veh Aβ VtA 1 10

3/3

β-actin

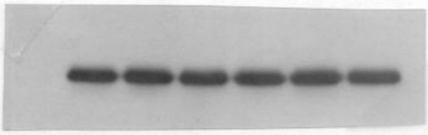

HSP 70 1° 1:1000 2° 1:5000

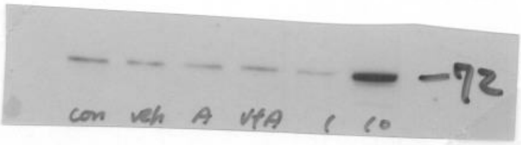

P25 1° 1:2000 2° 1:8000

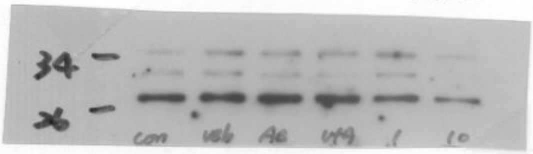

PHF 1° 1:1000 2° 1:5000.

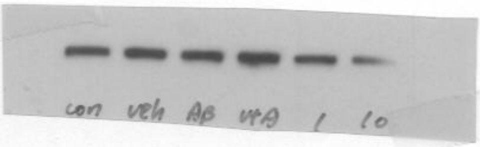

CDK5 1° 1:1000 2° 1:5000

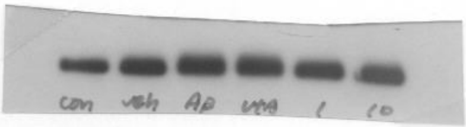

total-tau 1° 1:1000 2° 1:5000

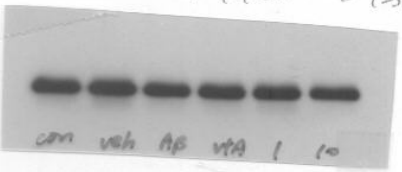

9/24

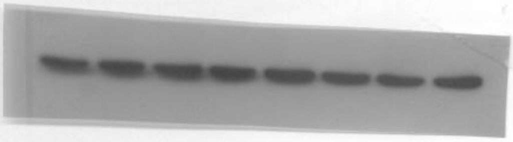

β- actin

1° 1:5000

2° 1:20000

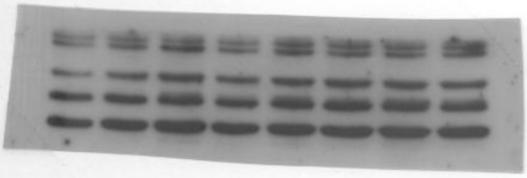

-34

-26

P35 - P25

1° 1:1000

2° 1:5000

con veh Aβ V+A 0.1 1 5 10

YC1

雜 band 很明顯

3/9 ODQ

β-actin

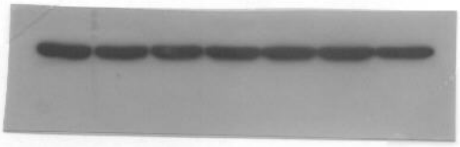

Calpain

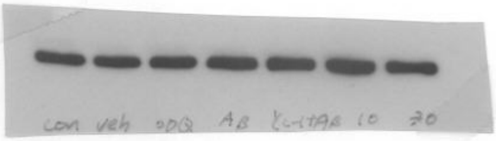

PHF-tau

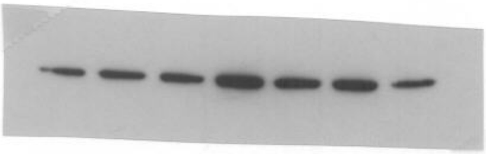

p35

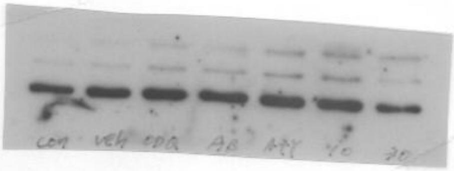

tau

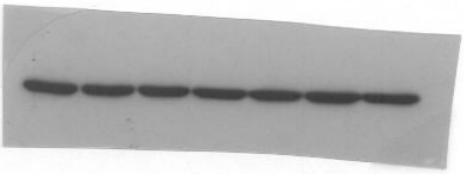

CDK5

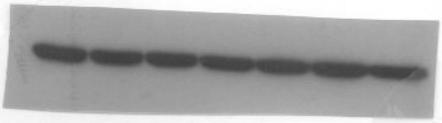

3/15 ODQ

β [illegible]tin

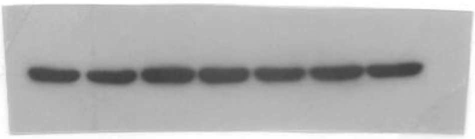

calpain

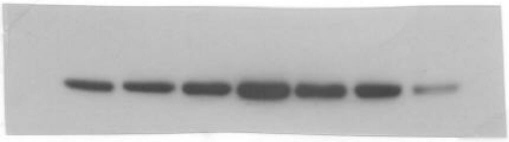

con | veh | ODQ 30 μM | Aβ | Aβ + YC-1 | Aβ + YC-1 + ODQ 10 μM | Aβ + YC-1 + ODQ 30 μM

PHF-tau

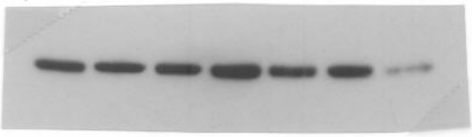

p35

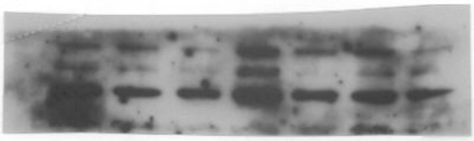

con | veh | ODQ | Aβ | Aβ + YC-1 | ODQ 10 μM | 30 μM ODQ

tau

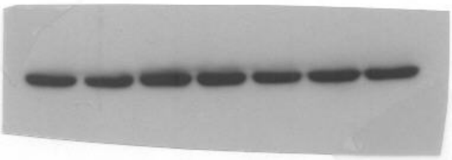

CDK5

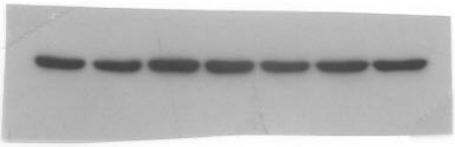

ODQ 10 μM, 20 μM

β-actin 1° 1:5000 2° 1:20000

43 –

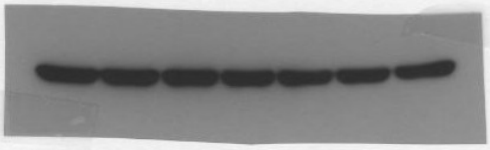

calpain 1° 1:500 2° 1:5000

80 –

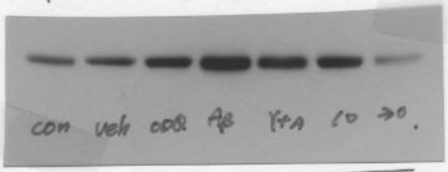

ODQ + Aβ + YC-1

p25 1° 1:2000 2° 1:8000

34 –

26 –

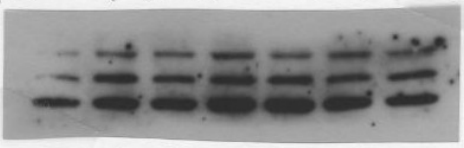

con veh ODQ Aβ Y+A 10 20

CDK5 1° 1:1000 2° 1:5000

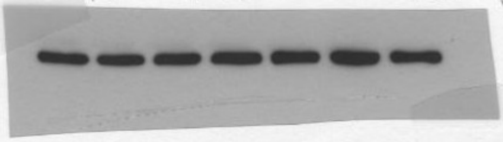

4/5 ODQ - tau

actin 1° 1:5000 2° 1:20000

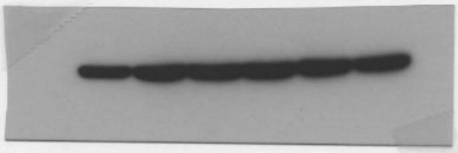

P-tau 1° 1:1000 2° 1:5000

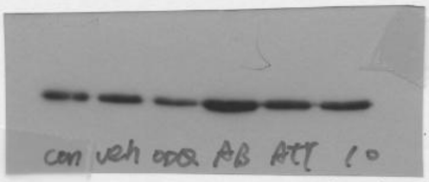

(ATT+ODQ)

total - tau

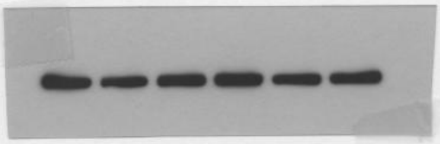

4/7 ODQ 10 μM

β-actin

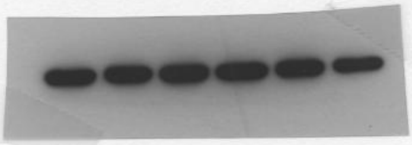

Calpain 1° 1:500 2° 1:5000

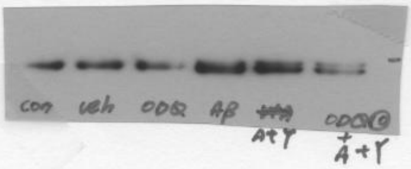

P35/25 1° 1:1000 2° 1:10000

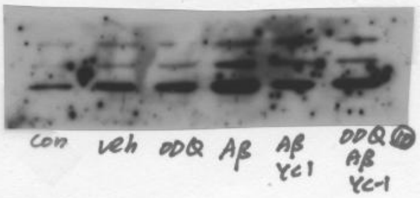

con veh ODQ Aβ Aβ YC1 ODQ⑩ Aβ YC-1

CDK5 1° 1:1000 2° 1:5000

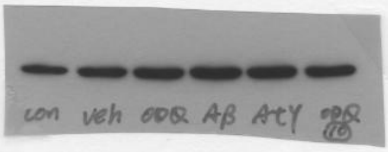

P-tau 1° 1:1000 2° 1:5000

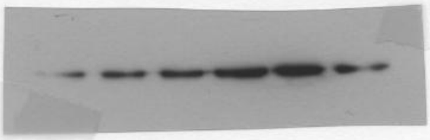

total-tau 1° 1:1000 2° 1:5000

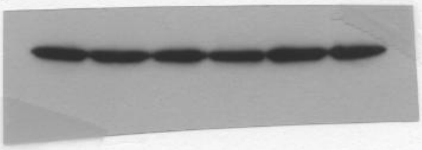

4/8

ODQ 10 μM

β-actin

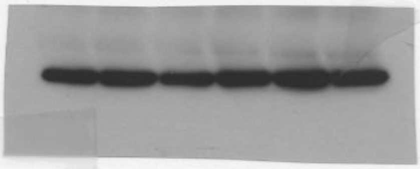

calpain 1° 1:500 2° 1:5000

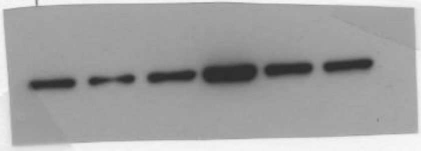

P-tau

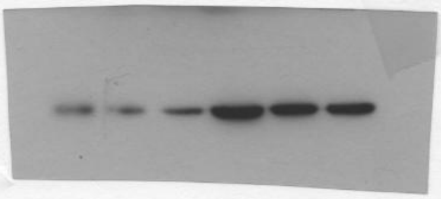

Q

P35/25 1° 1:2000 2° 1:8000

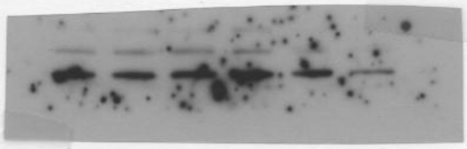

total-tau

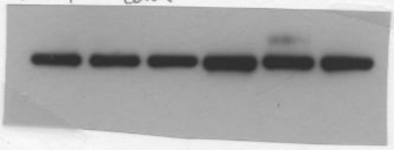

Supplement: Figure S1 — The raw data for the Figure 3D, 3F , and Figure 4 . Protein samples from PC12 cells were electrophoresed and then transferred to nitrocellulose membranes. The nitrocellulose membrane was cut according the molecular weight of protein and be incubated with different protein antibody. Therefore, one result of different proteins could get in one nitrocellulose membrane. Meanwhile, these proteins had the same internal standard (β-actin). (PDF) [file pone.0069320.s001.pdf]
